# Effect of exercise rehabilitation training on cardiac function and quality of life after PCI for acute myocardial infarction

**DOI:** 10.3389/fcvm.2025.1662376

**Published:** 2025-12-10

**Authors:** Jianru Zhou, Li Cai, Wei Zhang, Zhigang Chen, Chunhui Huang, Yonghong Zheng, Yiping Shi, Jianfeng Shen

**Affiliations:** 1 Department of Cardiovascular Medicine, Liyang Hospital of Traditional Chinese Medicine, Liyang, Jiangsu, China; 2 Department of Operating Room, Liyang People’s Hospital, Liyang, Jiangsu, China

**Keywords:** exercise rehabilitation training, acute myocardial infarction, percutaneous coronary intervention, cardiac function, inflammatory factor, quality of life

## Abstract

**Objective:**

The aim of this study was to investigate the effects of exercise rehabilitation training on left ventricular ejection fraction (LVEF) and quality of life in patients after percutaneous coronary intervention (PCI) for acute myocardial infarction (AMI).

**Methods:**

This study included 180 patients with AMI who underwent PCI between January 2022 and February 2024, and the patients were divided into control group (*n* = 90) and exercise group (*n* = 90) according to the randomized numeric table method. The control group was given routine care, and the exercise group was given rehabilitation exercise training on the basis of the control group. The rehabilitation exercise training lasted for 3 months, covering the inpatient rehabilitation period and the post-discharge rehabilitation period, with low-intensity bed and bedside activities as the main focus during the inpatient period, and aerobic exercise combined with strength and flexibility training as the main focus after discharge. The primary endpoint was LVEF at 3 months. Key secondary endpoints included exercise capacity [6-minute walk distance (6MWD)], quality of life [36-Item Short Form Health Survey (SF-36)], biomarkers of inflammation/oxidative stress, and adverse cardiovascular events.

**Results:**

Baseline characteristics were similar between groups (*P* > 0.05). After 3 months, LVEF improved in both groups, with a greater increase in the exercise group (between-group *P* ≤ 0.01). Exercise rehabilitation also improved 6MWD and SF-36 domains and reduced adverse cardiovascular events [8/90 (8.9%) vs. 18/90 (20.0%); risk ratio 0.44; 95% confidence interval (CI) 0.20–0.97; *P* = 0.034].

**Conclusion:**

Adding exercise rehabilitation to usual care after PCI for AMI improved LVEF and key clinical outcomes. These findings support routine integration of structured exercise rehabilitation in post-PCI care.

## Introduction

Acute Myocardial Infarction (AMI) is a common and serious coronary artery disease characterized by stenosis or occlusion of coronary arteries further leading to hypoxia and ischemia of myocardial tissue (1). In recent years, its morbidity and mortality are at a high level globally with the changes in lifestyle and the acceleration of population aging (2). Despite significant advances in medical technology in recent years, Percutaneous Coronary Intervention (PCI) has become one of the mainstays of treatment for AMI by virtue of its significant advantage of being able to rapidly unblock infarct-related vessels and restore myocardial perfusion in a timely manner (3, 4).

However, PCI is not the end point of treating AMI, PCI can only temporarily relieve the mechanical stenosis or occlusion of coronary arteries, and the physiopathological process of atherosclerosis in patients does not terminate after PCI. In addition, the sudden onset of AMI can bring a double blow to the patient's body and psychology, and the decline in cardiac function, decreased exercise endurance, and malignant arrhythmia can occur after the operation, which affects the patient's life quality and prognosis (5–7). With the continuous development of medical science and technology, people have a more profound knowledge of cardiovascular diseases and gradually become dissatisfied with the traditional clinical treatment, and it may be difficult to fully meet the rehabilitation needs of patients relying only on medication and routine care (6, 8). Cardiac rehabilitation is based on measures designed to help patients minimize recovery time after a cardiac event and maximize physical, social, and psychological performance, aiming to promote health behaviors and improve patient health and outcomes (9, 10).

Physiologically, the heart can adapt to chronic exercise to meet the body's increased oxygen demand, a process known as “remodeling” (11, 12). A growing number of studies have shown that exercise rehabilitation training, as an effective adjunctive therapy, has a significant role in improving cardiac function, psychological status, and quality of life in patients with cardiovascular disease (13). For example, a meta-analysis of the effects of exercise training on left ventricular remodeling after myocardial infarction showed that exercise training had a beneficial effect on left ventricular remodeling in clinically stable postmyocardial infarction patients, with the greatest benefit when training was initiated early after myocardial infarction (starting at 1 week) and continued for more than 3 months (14). Early exercise-based rehabilitation is effective in improving cardiorespiratory fitness, health-related quality of life and functional capacity after AMI (15, 16).

Based on the above background, this study aims to systematically assess the effects of exercise rehabilitation on cardiac function and quality of life in patients with AMI after PCI, to further explore the underlying mechanisms, and to provide a scientific basis for clinical practice. Specifically, we will compare the differences between exercise rehabilitation and conventional care in improving patients' cardiac function and quality of life through a randomized controlled trial design, with the aim of providing new ideas for optimizing postoperative rehabilitation strategies for AMI patients.

## Materials and methods

**Figure 1:**
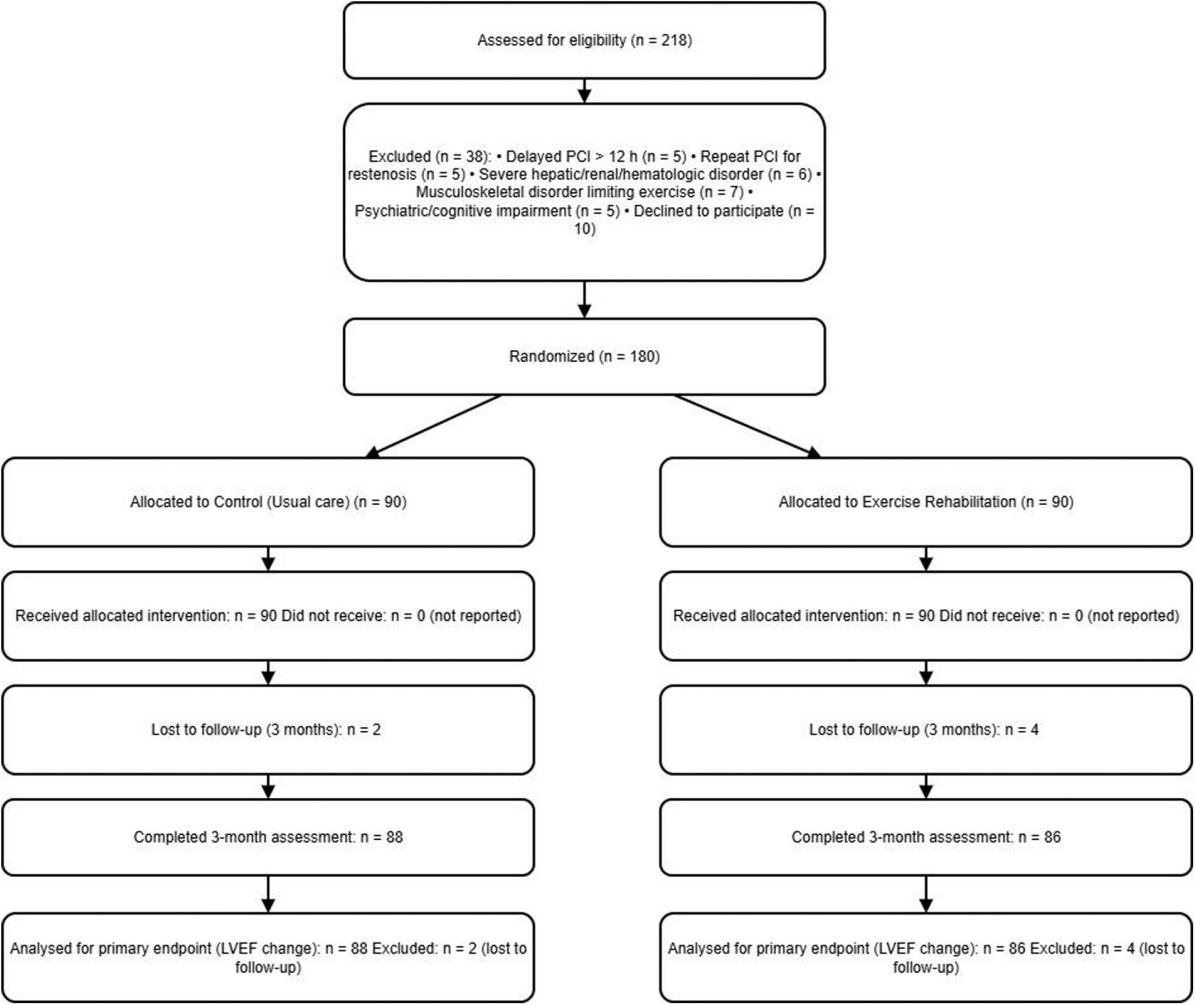
Subject flowchart.

The study flowchart is provided as Figure 1.

### Study population

218 AMI patients admitted to our cardiology department who underwent PCI from January 2022 to February 2024 were collected. Eligible patients provided written informed consent and were randomized within 24–48 h after PCI once hemodynamic stability was confirmed (no recurrent chest pain or ongoing ischemia, no vasoactive support, Killip class ≤ II). Randomization occurred before any rehabilitation exercise. Follow-up and outcome ascertainment began at randomization. Eligible, clinically stable patients were randomized 1:1 to usual care (control) or exercise rehabilitation in addition to usual care. The study was approved by the Ethics Committee of our hospital and complied with the Declaration of Helsinki.

### Inclusion and exclusion criteria

Inclusion criteria: 1. in accordance with the Guidelines for the Diagnosis and Treatment of Acute ST-segment Elevation Myocardial Infarction and confirmed by electrocardiogram (ECG); 2. Successful primary PCI within 12 h of symptom onset; 3. Clinically stable within 24–48 h post-PCI (no recurrent chest pain or ongoing ischemia, no vasoactive/inotropic support, Killip class ≤ II). voluntarily participating in this study and signing an informed consent form, and being able to cooperate with the training and regular review after discharge from the hospital.

Exclusion criteria: 1. Non–ST-elevation myocardial infarction (NSTEMI) or unstable angina as the index presentation; 2. Repeat PCI before randomization (in-hospital re-intervention after the index AMI PCI) or in-stent restenosis (ISR)–related AMI as the index event requiring target-lesion revascularization; 3. Severe arrhythmia and undergoing heart transplantation, heart valve replacement, pacemaker implantation and other surgeries; 4. Severe comorbid systemic diseases, defined as: Liver dysfunction: alanine aminotransferase (ALT) or aspartate aminotransferase (AST) > 3× upper limit of normal (ULN), or total bilirubin > 2× ULN; Renal dysfunction: estimated glomerular filtration rate (eGFR) < 60 mL/min/1.73 m^2^; Hematopoietic disorders: hemoglobin < 9 g/dL, platelets < 100 × 10⁹/L, or leukocytes < 3.0 × 10⁹/L; Severe psychiatric illness: active psychosis, major depression, or cognitive impairment that limits adherence to rehabilitation; 5. Musculoskeletal disorders (fracture, osteoarthritis, synovitis, or other conditions) limiting safe participation in exercise; 6 Combined with psychiatric diseases, cognitive dysfunction. Functional disorders.

#### Primary endpoint

Change in left ventricular ejection fraction (LVEF, %) from baseline (post-PCI, pre-randomization) to 3 months, assessed by transthoracic echocardiography following standard recommendations.

#### Secondary endpoints

Fractional shortening (FS), left ventricular end-diastolic diameter (LVEDD), left ventricular end-systolic diameter (LVESD), 6-minute walk distance (6MWD), 36-Item Short Form Health Survey domain scores (SF-36); inflammatory markers—high-sensitivity C-reactive protein (hs-CRP), interleukin-6 (IL-6), tumor necrosis factor-α (TNF-α), soluble intercellular adhesion molecule-1 (sICAM-1); oxidative stress markers—superoxide dismutase (SOD), total antioxidant capacity (T-AOC), catalase (CAT), malondialdehyde (MDA), and a 3-month composite of adverse cardiovascular events.

### Sample size estimation

The trial was powered for the primary endpoint, change in left ventricular ejection fraction (LVEF) from baseline (post-PCI, pre-randomization) to 3 months. Based on prior exercise-based cardiac rehabilitation literature and local variability, we assumed a between-group difference in mean LVEF change of *Δ* = 4 percentage points with a standard deviation of change *σ* = 8. Using a two-sided *α* = 0.05% and 90% power, the required sample size per group for a parallel-group comparison is:

$$n\;=\;\frac{2{\left(Z_{1-\frac{\alpha }{2}}+\;Z_{1-\beta }\right)}^{2}\sigma^{2}}{\Delta^{2}}$$

which with $Z_{1-0.025}=1.96$, $Z_{1-0.10}=1.28$, *σ* = 8, and *Δ* = 4 gives *n* ≈ 84 per group (total ≈ 168). Allowing for ∼5%–7% attrition, we pre-specified a target of 90 per group (total 180) and enrolled accordingly.

### Treatment

Nursing measures in the control group: patients in the control group were given conventional drug therapy, including antiplatelet aggregation (e.g., aspirin combined with clopidogrel or tegretol), statin lipid modulation (e.g., atorvastatin, rasuvastatin), β-blockers (e.g., metoprolol, bisoprolol), angiotensin-converting enzyme inhibitors (ACEIs), or angiotensin II receptor antagonists (ARBs) (e.g., enalapril, valsartan) and other drugs to improve myocardial ischemia, prevent thrombosis, and control blood pressure and blood lipids. At the same time, routine nursing interventions were carried out, including close monitoring of vital signs, dietary guidance (low-salt, low-fat, low-sugar diet), rest and activity guidance (absolute bed rest during the acute stage, gradually increasing the amount of activity), psychological care, etc. Routine health education was also carried out to inform patients of disease-related knowledge, drug intake methods and precautions.

Exercise group rehabilitation exercise training program: The exercise group, on the basis of treatment and care in the control group, implemented a 3-month exercise rehabilitation training program according to the different characteristics of the hospitalization rehabilitation period and the post-discharge rehabilitation period. The specific program is as follows:

1. Inpatient rehabilitation period: patients’ postoperative conditions are relatively unstable and their bodies are relatively weak, so the main purpose of the rehabilitation exercise at this stage is to promote the initial recovery of body functions, enhance the patients’ ability to adapt to exercise, and at the same time ensure the safety of exercise.
2. Exercise type: bed activities and bedside activities are the main activities. Bedside activities include passive and active activities of the limbs, such as flexion and extension of the joints of the limbs, slow flexion and extension of each joint for 5∼10 times, 3∼4 groups per day, which can promote the blood circulation of the limbs and prevent muscle atrophy and joint stiffness; deep breathing training, the patient takes a comfortable position, slowly inhale to make the abdomen bulge, and then slowly exhale, each training for 5∼10 min, 3∼4 times per day, which can help to improve the pulmonary This will help improve the ventilation function of the lungs. Bedside activities start from sitting up training, the patient gradually sits up from the lying position with the assistance of medical and nursing staff, and then conducts bedside standing training after adapting to it, and the time for standing up starts from 5∼10 min each time, and is gradually prolonged according to the patient's tolerance situation, and is conducted 2∼3 times a day.

#### Exercise intensity

During the inpatient phase, exercise intensity was maintained at a low level to ensure safety and gradual recovery. Intensity was prescribed according to the patient's tolerance, targeting an increase of ≤20 beats/min from resting heart rate, corresponding approximately to ≤40% of heart rate reserve (HRR), 2–3 METs, or an RPE of 9–11 on the Borg 6–20 scale, as recommended by the AHA and ESC guidelines for early cardiac rehabilitation. Exercise was terminated if palpitations, chest tightness, or excessive fatigue occurred, and vital signs were continuously monitored.

#### Exercise time

The total duration of each exercise should be controlled at 10∼15 min, and can be gradually increased to about 20 min with the improvement of the patient's physical condition. The exercise process is divided into preparatory activities (2∼3 min, such as simple body relaxation activities), formal exercise (5∼12 min) and relaxation activities (3∼5 min, such as slow deep breathing and muscle relaxation exercises).

#### Exercise frequency

2∼3 times of exercise training per day, and the number of training sessions can be increased appropriately if tolerated by the patient's body.

1. Post-discharge rehabilitation period: the patient's body functions have been recovered to some extent, and at this stage, on the basis of ensuring safety, the intensity and difficulty of exercise are gradually increased to further improve cardiopulmonary function and exercise endurance, and to promote the recovery of cardiac function and the enhancement of quality of life.

#### Exercise type

Aerobic exercise, combined with appropriate amount of strength training and flexibility training. Aerobic exercise includes walking, choosing a place with fresh air and flat ground, walking at a moderate pace (about 60∼80 steps per minute) and gradually transitioning to fast walking (about 100∼120 steps per minute); jogging, starting to try when the patient's ability to walk is good and there is no discomfort, paying attention to maintaining a smooth rhythm and breathing; cycling, choosing to ride outdoors or use indoor fitness bike. Riding speed is adjusted according to the patient's own condition. Strength training using elastic bands or small weight dumbbells, such as arm flexion and extension, shoulder abduction for the upper limbs, leg extension for the lower limbs, leg lifting and other exercises, each action is performed 8∼12 times, each interval of 1∼2 min, each time 2∼3 groups, in order to enhance muscle strength and improve the body's metabolic rate. Flexibility training through simple stretching movements, such as neck, waist and leg stretching, hold each movement for 15∼30 s, perform 2∼3 groups to improve joint mobility and prevent sports injuries. Exercise intensity: The heart rate reserve (HRR) method combined with conscious exertion rating (RPE) was used to determine the exercise intensity. Exercise intensity was initially set at 40%–60% of HRR, which corresponded to an RPE score of 11–13 (slightly strenuous), and then gradually increased to 60%–80% of HRR, with an RPE score of 13–15 (strenuous), as the patient's exercise endurance improved. For example, a 50-year-old patient with a maximal heart rate of approximately 170 beats per minute (220 - age), a resting heart rate of 70 beats per minute, and a heart rate reserve of 100 beats per minute would have a target heart rate range of (100 × 40% + 70) = 110 beats per minute to (100 × 60% + 70) = 130 beats per minute during initial exercise.

#### Exercise duration

The total duration of each exercise is 30∼45 min, including 5∼10 min of warm-up exercises (e.g., slow walking, joint mobilization exercises), 20∼30 min of formal exercise (a combination of aerobic exercise, strength training, and flexibility training), and 5∼10 min of relaxation exercises (e.g., slow walking, stretching of muscles).

#### Exercise frequency

Exercise frequency is 3–5 times per week to ensure that patients have enough rest time for recovery. During exercise, patients should carry first aid medication, such as nitroglycerin, and inform their families of the exercise precautions so that they can deal with any discomfort in time. At the same time, patients are advised to go to the hospital for regular review, and according to the review results, the professional physician will adjust the exercise rehabilitation program in a timely manner.

### Data collection

Patients' age, gender, Body Mass Index (BMI), smoking history, alcohol consumption history, comorbidities (hypertension, hyperlipidemia, diabetes mellitus), infarct locations (anterior wall, inferior wall, posterior-inferior wall, lateral wall), cardiac function class (I∼III), number of stents (≤1, ≥2) were collected. wall), cardiac function class (I-III), and number of stents (≤1, ≥2).

### Clinical assessment

Cardiac function, exercise tolerance, inflammatory factor levels, oxidative stress indicators, and quality of life were compared between the two groups before and 3 months after the intervention. And count the adverse cardiovascular events such as angina pectoris, re-infarction, heart failure, arrhythmia and so on.

#### Cardiac function indexes

Vivid-7Demision color echocardiography from GE was used to measure the cardiac function of coronary artery disease patients in the two groups, including LVEF, LVEDD, LVESD, and left ventricular short-axis shortening.

#### Exercise endurance indexes

Patients' exercise endurance was rated by using the 6 min walking test, patients walked on a flat, unobstructed corridor in the room, and the distance they were able to walk in 6MWD.

#### Inflammatory factor indexes

Before and 3 months after the intervention, patients were collected 5 mL of fasting venous blood in the early morning, centrifuged at 3,000 r/min for 10 min, serum was separated and stored in the refrigerator at - 80℃ for measurement. The serum was separated and stored at −80℃ in a refrigerator for measurement. hs-CRP, IL-6, TNF-α, and soluble intercellular adhesion molecule-1 (sICAM-1) were measured by enzyme-linked immunosorbent assay (ELISA) according to the manufacturers' instructions (hs-CRP kit E-EL-H5134, Wuhan Eliot Biotechnology; IL-6 PI325, TNF-α PT518, and sICAM-1 PI498, Shanghai Biyuntian Biotechnology). All operations were performed in strict accordance with the instructions.

#### Indicators of oxidative stress

The SOD Activity Test Kit (S0101S), T-AOC Test Kit (A015-1-2), CAT Assay Kit (A007-1-1) were used to determine the activity of SOD, T-AOC, and CAT in serum. A malondialdehyde (MDA) assay kit (A003-1-1) was used to determine the serum MDA content. The SOD kit was purchased from Shanghai Biyuntian Biotechnology Co., Ltd, and the T-AOC, CAT and MDA kits were purchased from Nanjing Jiancheng Bioengineering Institute. All the operations were carried out in accordance with the instructions.

#### Quality of life assessment

The quality of life of patients was assessed by using the Short Form Health Survey (SF-36), which contains the following scales: General health, Physical functioning, Role physical, Bodily pain, Role emotional, Vitality and Vitality. emotional, Vitality, Social functioning and Mental health. Each dimension is scored on a scale ranging from 0 to 100, with higher scores indicating better quality of life (17).

#### Incidence of adverse cardiovascular events

The adverse cardiovascular events such as angina pectoris, re-infarction, heart failure, arrhythmia and so on were counted in both groups.

### Statistical analysis

Data were statistically analyzed and graphed using GraphPad Prism 9.5.0 software (GraphPad Software Inc., San Diego, CA, USA). The Shapiro–Wilk test was used to test for normal distribution, and measures that conformed to normal distribution were expressed as mean ± standard deviation, with independent samples t-tests for comparisons between the two groups, and paired samples t-tests for comparisons between pre- and post-intervention within groups. Measurement information that did not conform to normal distribution was expressed as quartiles, Mann–Whitney U test was used for comparison between two groups, and Wilcoxon Signed Rank Test was used for pre- and post-intervention comparisons. Count data were expressed as number of cases and percentages, and comparisons between groups were made using the chi-square test. *p* was a two-sided test, and differences were considered statistically significant at *p* < 0.05.

## Results

### Comparison of baseline data between the two groups

One hundred and eighty patients with AMI who were admitted at our hospital and underwent PCI from 12 January 2022 to February 2024 were selected for the study. A total of 218 patients were screened after PCI during the study period; 38 were excluded (delayed PCI >12 h after symptom onset, *n* = 5; repeat PCI for restenosis, *n* = 5; severe hepatic/renal dysfunction or hematologic disorder, *n* = 6; fractures/osteoarthritis or other musculoskeletal disease, *n* = 7; psychiatric disorder or cognitive impairment, *n* = 5; declined participation, *n* = 10). One hundred eighty (180) patients were randomized and allocated equally to the control group (*n* = 90) and the exercise group (*n* = 90). The control group was given routine care, and the exercise group was given rehabilitation exercise training on the basis of the control group. Of 180 randomized participants, 6 were lost to follow-up by 3 months (2/90 control; 4/90 exercise). Thus, 174/180 (96.7%) completed 3-month assessments. There was no significant difference between the two groups in terms of age, gender, Body Mass Index (BMI), smoking history, drinking history, family history of coronary artery disease, comorbidities (hypertension, hyperlipidemia, diabetes mellitus), Infarct locations, cardiac function classification and the number of stents put in, etc. (all *P* > 0.05), see Table 1.

**Table 1:** Comparison of baseline information between the two groups.

Sports event	Control group (*n* = 90)	Exercise group (*n* = 90)	*t*/*χ*^2^	*P*
Age (years)	51.29 ± 6.66	51.63 ± 7.27	0.331	0.741
Sex (m/f)	56/34	60/30	0.388	0.533
BMI（kg/m^2^）	24.67 ± 1.89	24.84 ± 2.03	0.393	0.561
Smoking history (n, %)	38 (42.22%)	43 (47.78%)	0.561	0.454
Drinking history (n, %)	30 (33.33%)	33 (36.67%)	0.220	0.6392
Comorbidities (n, %)				
High blood pressure	50 (55.56%)	55 (55.56%)	0.571	0.450
Hyperlipidemia	36 (40.00%)	40 (44.44%)	0.364	0.546
Diabetes	22 (24.44%)	25 (27.78%)	0.259	0.611
Infarct locations (*n*, %)				
Anterior wall	35 (38.89%)	37 (41.11%)	0.467	0.926
Inferior wall	29 (32.22%)	27 (30.00%)		
Side wall	18 (20.00%)	16 (17.78%)		
Others	8 (8.89%)	10 (11.11%)		
Killip classification (*n*, %)				
I	38 (42.22%)	43 (47.78%)	0.563	0.755
II	33 (36.67%)	30 (33.33%)		
III	19 (21.11%)	17 (18.89%)		
Number of stents (*n*, %)				
≤1	50 (55.56%)	56 (62.22%)	0.826	0.3634
≥2	40 (44.44%)	34 (37.78%)		

BMI, body mass index. Shapiro–Wilk test was used to test for normal distribution, and measurements conforming to normal distribution were expressed as mean ± standard deviation, and comparisons between two groups were made using the independent samples t-test. Count data were expressed as number of cases and percentage, and the chi-square test was used for comparison between groups. The difference was considered statistically significant at *P* < 0.05.

### Comparison of cardiac function between two groups of patients

**Figure 2:**
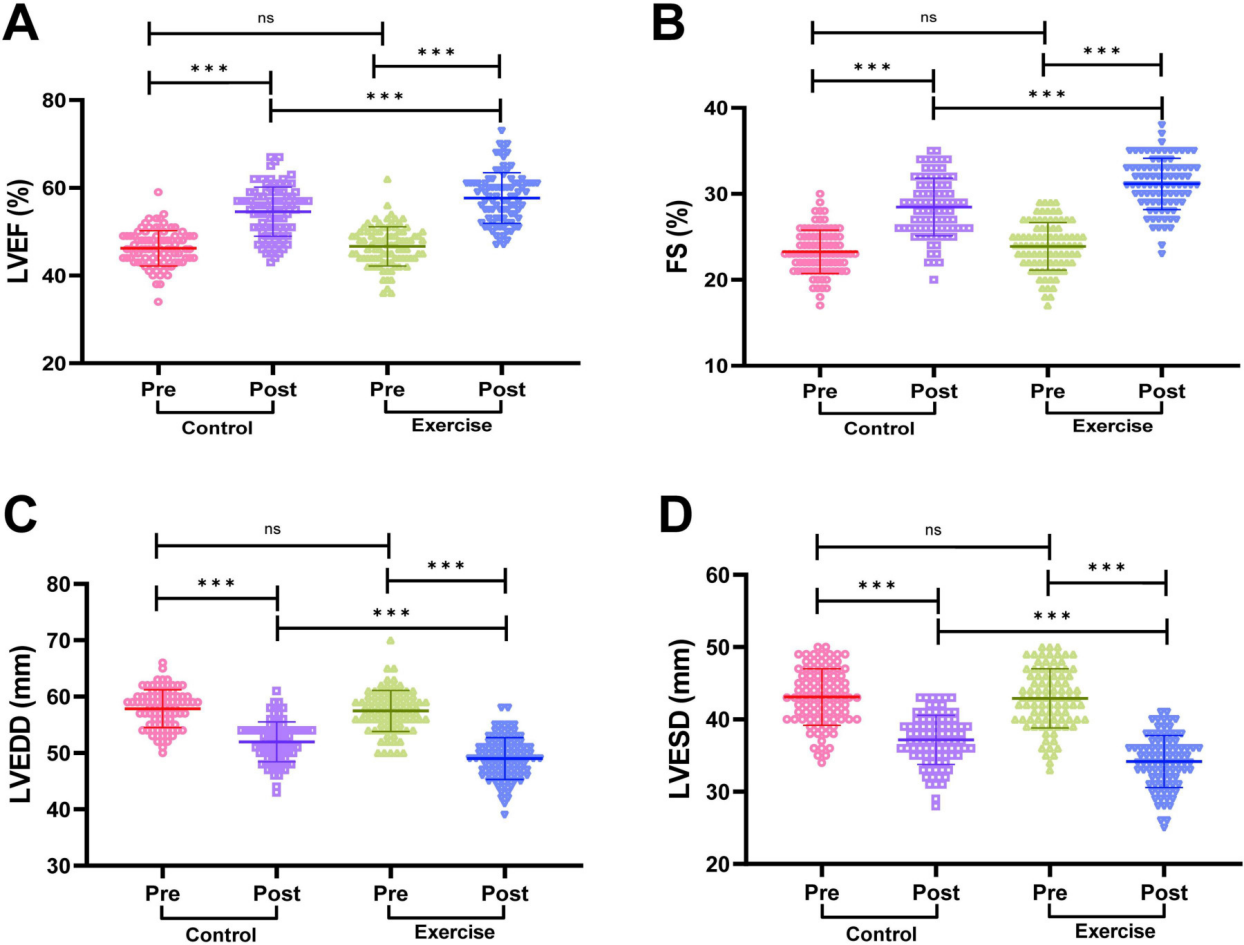
Comparison of cardiac function between the two groups. **(A)** Left ventricular ejection fraction (LVEF). **(B)** Left ventricular end-diastolic dimension (LVEDD). **(C)** Left ventricular end-systolic dimension (LVESD). **(D)** Fractional shortening (FS). Notes: The Shapiro-Wilk test was used to assess normality. Normally distributed data are expressed as mean ± standard deviation. Between-group comparisons used independent samples *t*-tests; within-group pre/post comparisons used paired *t*-tests. Significance levels: ns (*P* > 0.05), **P < 0.001.

We compared the cardiac function of patients in the two groups, and the results are shown in Figure 2. The cardiac function indexes of patients in the two groups before the intervention were not statistically significant (both *P* > 0.05); the LVEF and FS of patients in the exercise group were significantly higher than those before the intervention, and the LVEDD and LVESD were significantly lower than those before the intervention (both *P* < 0.001); moreover, the LVEF and FS of patients in the exercise group were significantly higher than those in the control group after the intervention. In addition, LVEF and FS were significantly higher in the exercise group than in the control group after the intervention, and LVEDD and LVESD were significantly lower than in the control group, with statistically significant differences (all *P* < 0.001). It is suggested that exercise rehabilitation training can more effectively improve the cardiac functional status of AMI patients after PCI.

### Comparison of exercise endurance indexes between two groups of patients

**Figure 3:**
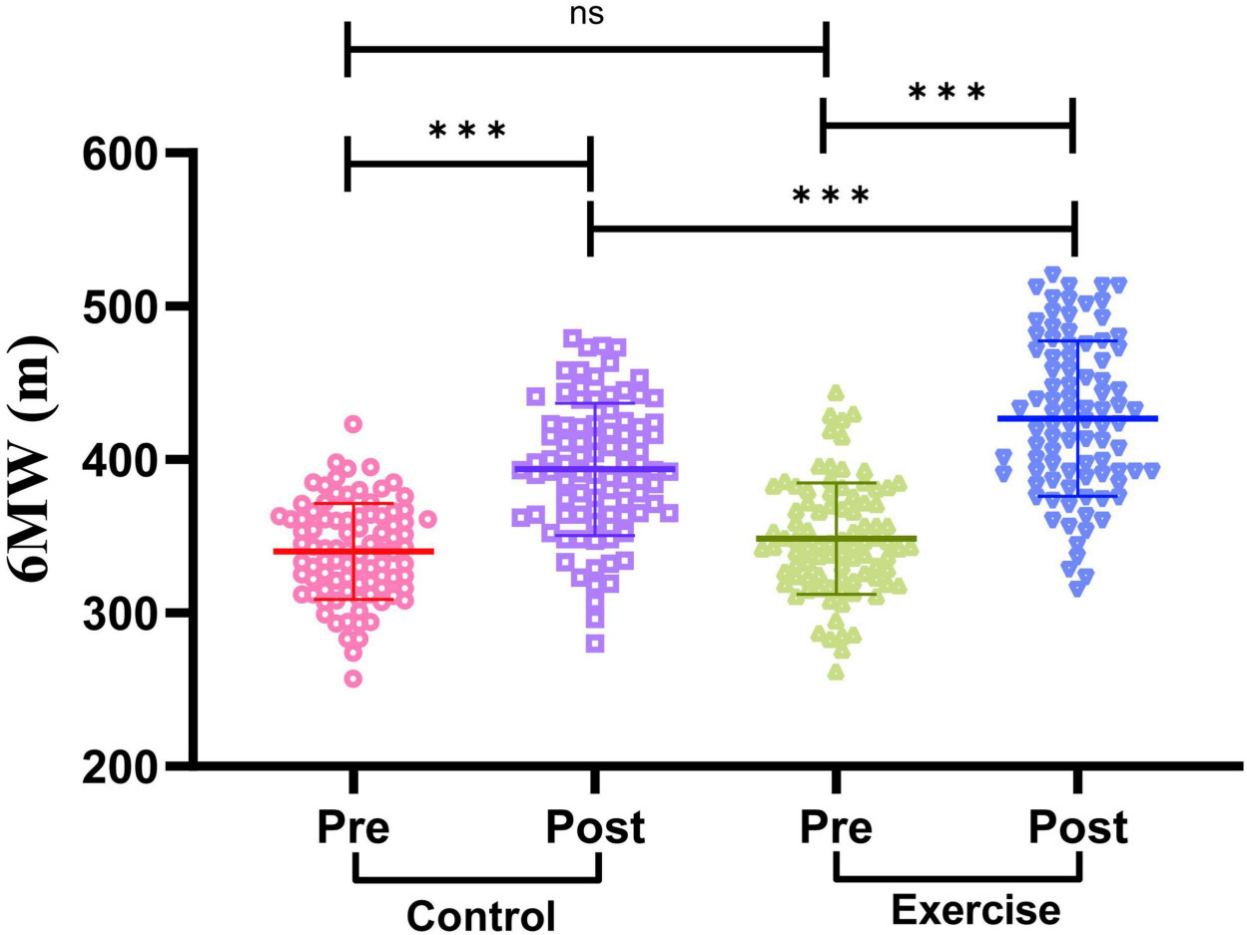
Comparison of exercise endurance indexes between two groups of patients. 6MWD, 6-minute walking distance. The Shapiro–Wilk test was used to test for normal distribution. Measures that conformed to normal distribution were expressed as mean ± standard deviation, and comparisons between the two groups were made using the independent samples t-test, while comparisons between the pre-intervention and post-intervention periods within the groups were made using the paired samples t-test. Differences were considered statistically significant at *P* < 0.05. ns indicates *P* > 0.05 and *** indicates *P* < 0.001.

We compared the exercise endurance of the two groups of patients before and after the intervention by 6MWD. The results are shown in Figure 3: Before the intervention, there was no significant difference in 6MWD between the two groups of patients (*P* > 0.05). After the intervention, the 6MWD of patients in both groups was significantly higher than that before the intervention (*P* < 0.001). In addition, the 6MWD of the exercise group was significantly higher than that of the control group after intervention (*P* < 0.001). It is suggested that exercise rehabilitation training has a significant effect on enhancing the exercise endurance of AMI patients after PCI.

### Comparison of inflammatory factor levels between the two groups of patients

**Figure 4:**
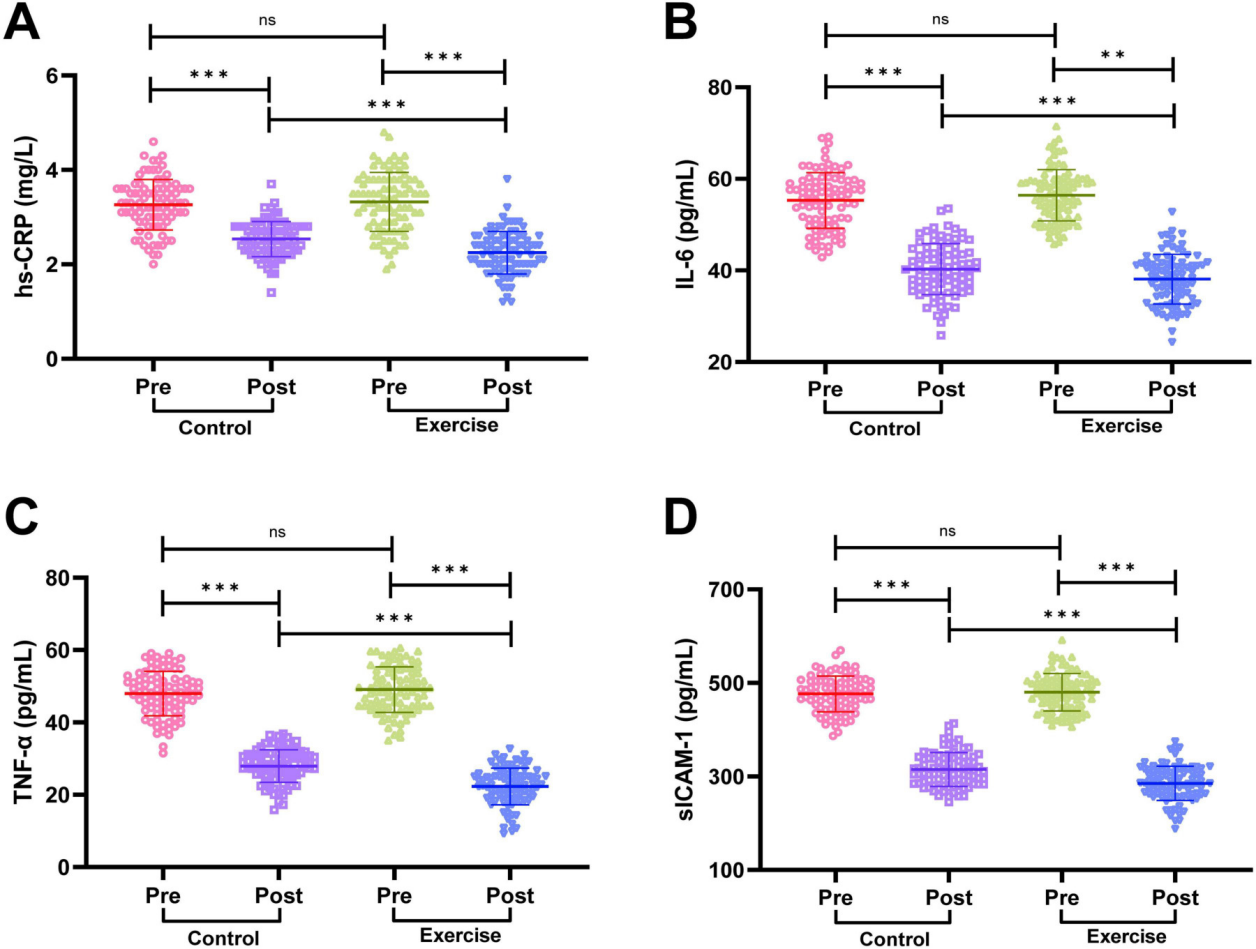
Comparison of inflammatory factor levels between the two groups. **(A)** High-sensitivity C-reactive protein (hs-CRP). **(B)** Interleukin-6 (IL-6). **(C)** Tumor necrosis factor-α (TNF-α). **(D)** Soluble intercellular adhesion molecule-1 (sICAM-1). Notes: Normality was tested with the Shapiro-Wilk test. Normally distributed data are shown as mean ± SD; between-group comparisons used independent t-tests. Significance levels: ns (*P* > 0.05), **P < 0.01, **P < 0.001.

We detected the serum levels of hs-CRP, IL-6, TNF-α and sICAM-1 by ELISA, and the results are shown in Figure 4. Before the intervention, there was no statistically significant difference in the serum levels of hs-CRP, IL-6, TNF-α and sICAM-1 in the two groups of patients (all *P* > 0.05). The serum levels of hs-CRP, IL-6, TNF-α and sICAM-1 were significantly lower in both groups after intervention (all *P* < 0.001), and the serum levels of hs-CRP, IL-6, TNF-α and sICAM-1 in the exercise group were significantly lower than those in the control group after intervention (all *P* < 0.01). It is suggested that exercise rehabilitation training can effectively reduce the level of inflammatory factors in AMI patients after PCI, so as to better regulate the inflammatory response in patients.

### Comparison of oxidative stress indicators between the two groups of patients

**Figure 5:**
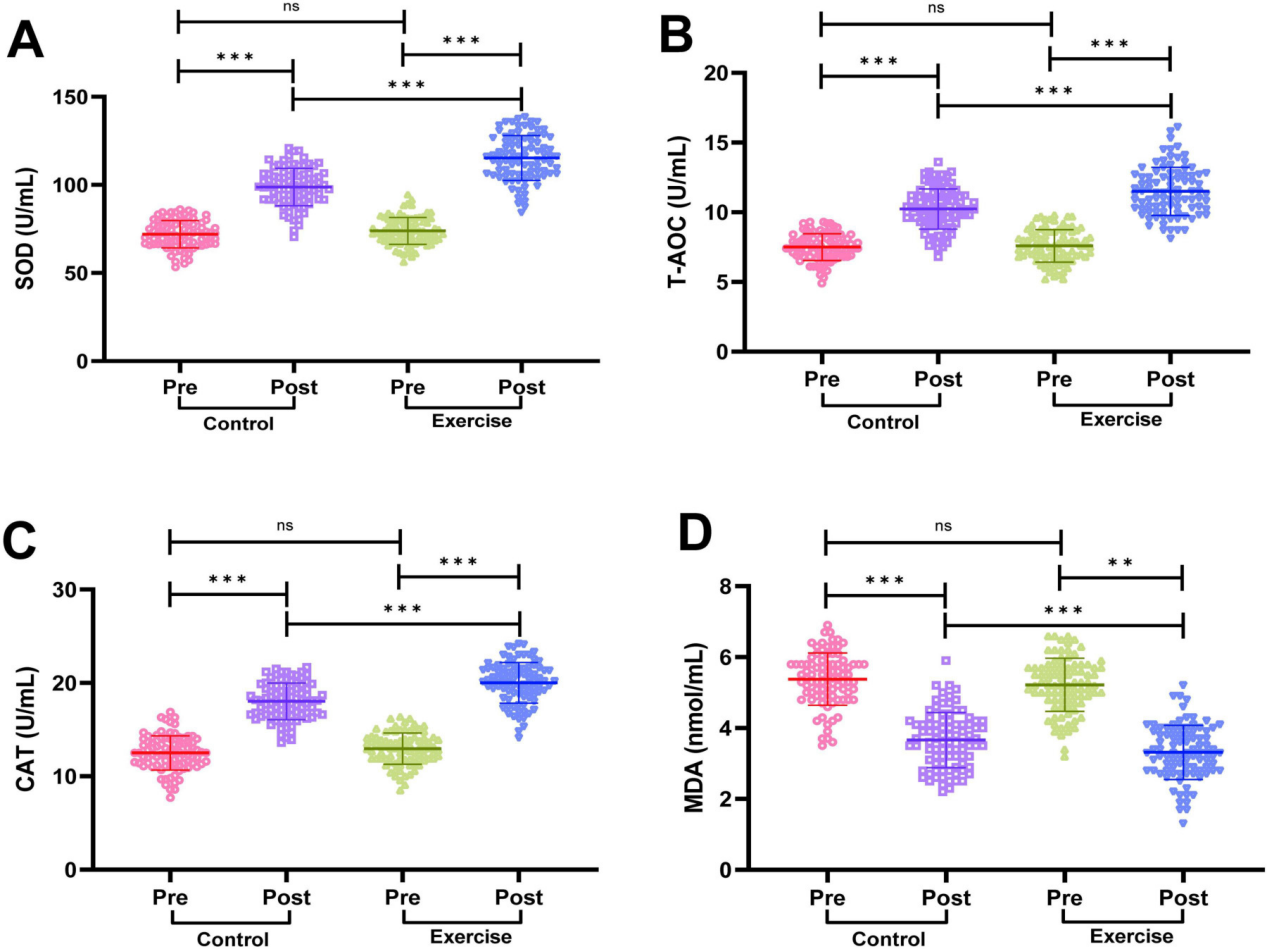
Comparison of oxidative stress indicators between the two groups. **(A)** Superoxide dismutase (SOD). **(B)** Total antioxidant capacity (T-AOC). **(C)** Catalase (CAT). **(D)** Malondialdehyde (MDA). Notes: Shapiro-Wilk test confirmed normality for parametric data (mean ± SD). Between-group comparisons used independent *t*-tests. Significance levels: ns (*P* > 0.05), **P < 0.01, **P < 0.001.

We measured serum SOD, T-AOC, CAT activity and MDA content by the kit, and the results are shown in Figure 5. Before the intervention, there was no statistically significant difference in serum SOD, T-AOC, CAT activity and MDA content in the two groups of patients (*P* > 0.05). After the intervention, serum SOD, T-AOC and CAT activities in the patients of the exercise group were significantly higher than before the intervention, while MDA content was significantly lower than before the intervention (*P* < 0.001); intergroup comparison revealed that SOD, T-AOC and CAT activities in the patients of the exercise group were significantly higher than that of the control group after the intervention, and MDA content was significantly lower than that of the control group (*P* < 0.001), suggesting that exercise rehabilitation training can significantly improve the oxidative stress status of AMI patients after PCI.

### Comparison of quality-of-life scores before and after intervention in both groups

We used the SF-36 Concise Health Questionnaire to evaluate patients' quality of life in terms of General health, Physical functioning, Role physical, Bodily pain, Role emotional, Vitality, Social functioning and Mental health aspects to evaluate the patients' quality of life, and it was found (Table 2) that before the intervention, the difference between the SF-36 scores of each dimension between the two groups was not statistically significant (all *P* > 0.05); after three months of intervention, the SF-36 scores of each dimension of the two groups were significantly higher (all *P* < 0.001); moreover, the scores of each dimension of the quality of life of patients in the exercise group were significantly higher than those of the control group after the intervention (all *P* < 0.05). It is suggested that exercise rehabilitation training can improve the quality of life of AMI patients after PCI.

**Table 2:** Comparison of quality of life scores before and after intervention in the two groups of patients.

Sports event	Timing	Control group (*n* = 90)	Exercise group (*n* = 90)	*t*	*P*
General health (Score)	Pre-intervention	58.61 ± 6.65	58.36 ± 7.48	0.242	0.809
	Post-intervention	71.57 ± 9.33	76.33 ± 9.75	3.350	0.001
	*t*	10.290	14.180		
	*P*	<0.001	<0.001		
Physical functioning (Score)	Pre-intervention	47.79 ± 6.16	48.91 ± 5.85	1.253	0.212
	Post-intervention	67.84 ± 7.65	72.13 ± 8.78	3.495	0.001
	*t*	19.110	20.260		
	*P*	<0.001	<0.001		
Role physical (Score)	Pre-intervention	51.27 ± 7.491	52.51 ± 7.420	1.120	0.264
	Post-intervention	68.63 ± 9.163	73.30 ± 9.533	3.348	0.001
	*t*	13.860	t = 15.570		
	*P*	<0.001	<0.001		
Bodily pain (Score)	Pre-intervention	41.50 ± 6.708	42.39 ± 7.069	0.865	0.388
	Post-intervention	63.60 ± 8.754	67.81 ± 9.087	3.166	0.002
	*t*	17.440	20.50		
	*P*	<0.001	<0.001		
Role emotional (Score)	Pre-intervention	61.43 ± 7.84	62.30 ± 9.08	0.685	0.494
	Post-intervention	75.62 ± 8.93	78.60 ± 9.04	2.223	0.028
	*t*	10.86	12.30		
	*P*	<0.001	<0.001		
Vitality (Score)	Pre-intervention	57.67 ± 6.84	58.88 ± 9.13	1.007	0.315
	Post-intervention	75.26 ± 9.93	79.62 ± 10.73	2.834	0.005
	*t*	14.280	13.730		
	*P*	<0.001	<0.001		
Social functioning (Score)	Pre-intervention	44.47 ± 6.76	46.08 ± 7.24	1.543	0.125
	Post-intervention	66.06 ± 8.52	70.21 ± 9.87	3.025	0.003
	*t*	18.780	17.370		
	*P*	<0.001	<0.001		
Mental health (Score)	Pre-intervention	62.22 ± 7.62	63.08 ± 8.15	0.727	0.468
	Post-intervention	78.28 ± 9.13	82.36 ± 9.51	2.934	0.004
	*t*	13.020	t = 14.540		
	*P*	<0.001	<0.001		

The Shapiro–Wilk test was used to test for normal distribution, and measures that conformed to normal distribution were expressed as mean ± standard deviation, and independent samples t-tests were used to compare the two groups, and paired t-tests were used before and after the intervention. Differences were considered statistically significant at *P* < 0.05.

### Comparison of adverse cardiovascular events between the two groups

The occurrence of adverse cardiovascular events (angina pectoris, re-infarction, heart failure, arrhythmia, etc.) during the intervention period was recorded in the two groups of patients, and the results showed that (Table 3): In the control group, there were three cases of angina pectoris, four cases of re-infarction, five cases of heart failure, and six cases of arrhythmia (total incidence 20.0%). In the exercise group, there were two cases of angina pectoris, two cases of re-infarction, two cases of heart failure, and three cases of arrhythmia (total incidence 8.9%). The overall incidence of adverse cardiovascular events was therefore significantly lower in the exercise group than in the control group (*χ*^2^ = 4.496, *P* = 0.034). The relative risk (RR) was 0.44, indicating that exercise rehabilitation reduced the risk of adverse cardiovascular events by 56%. The absolute risk reduction (ARR) was 11.1%, meaning that for every 100 patients receiving exercise rehabilitation, approximately 11 adverse events could be prevented. These findings suggest that exercise rehabilitation training after PCI for acute myocardial infarction significantly reduces the risk of subsequent adverse cardiovascular events.

**Table 3:** Comparison of adverse cardiovascular events between the two groups of patients.

Adverse cardiovascular events	Control group (*n* = 90)	Exercise group (*n* = 90)	χ^2^	*P*
Angina pectoris (*n*, %)	3 (3.33%)	2 (2.22%)		
Re-infarction (*n*, %)	4 (4.44%)	2 (1.11%)		
Heart failure (*n*, %)	5 (5.56%)	2 (2.22%)		
Arrhythmia (*n*, %)	6 (6.67%)	3 (3.33%)		
Total incidence (%)	18 (20.00%)	8 (8.89%)	4.496	0.034

Counting information was expressed as number of cases and percentage, and the chi-square test was used for comparison between groups. Differences were considered statistically significant at *P* < 0.05.

## Discussion

PCI can help the stenotic coronary arteries reopen, prompting the blood supply of myocardial tissues to return to normal and reduce ischemia, but routine postoperative care does not eliminate the risk factors of the disease, and patients are prone to anxiety, depression, and other adverse emotions due to restricted activities and other problems during bed rest, which is not conducive to the prognosis (18, 19). This study focused on the effects of exercise rehabilitation training on AMI patients after PCI, and the results showed that exercise rehabilitation training was effective in improving patients' cardiac function, exercise endurance, inflammatory factor levels, oxidative stress status, quality of life, and reducing the incidence of adverse cardiovascular events.

In terms of improving cardiac function, patients in the exercise group had significantly higher LVEF and FS, and significantly lower LVEDD and LVESD. This result is consistent with previous studies, which reported that exercise rehabilitation training can promote cardiac left ventricular remodeling, during which cardiac oxygen demand increases, and in order to satisfy the demand, myocardial contractility is enhanced, and cardiac structure and function are gradually optimized, thus enhancing cardiac function (14, 20, 21). Exercise also acts directly on the vascular system, improving endothelial and coronary smooth muscle function, enhancing coronary vasodilatation, providing a more adequate blood supply to the myocardium, and further supporting cardiac function recovery (22, 23).

For the improvement of exercise endurance, 6MWD was significantly increased in the exercise group. Low-intensity activities during inpatient rehabilitation helped patients adapt to exercise, and aerobic exercise, strength training and flexibility training after discharge gradually improved cardiopulmonary function and muscle strength. Aerobic exercise enhances cardiorespiratory endurance, strength training improves muscle metabolic rate and contraction capacity, and flexibility training ensures the fluidity and safety of exercise, and the three synergistic effects result in a significant increase in patients' exercise endurance (24, 25). Regular aerobic exercise can enhance cardiorespiratory function, improve the body's ability to take up, transport and utilize oxygen, while improving skeletal muscle metabolism, enhancing muscle strength and endurance, enabling patients to tolerate longer and more intense exercise (26).

Inflammatory response plays an important role in the development of cardiovascular disease (27). Studies have shown that exercise rehabilitation significantly reduces the levels of inflammatory factors, including hs-CRP, IL-6, TNF-α, and sICAM-1, in AMI patients after PCI. Exercise rehabilitation modulates the inflammatory response through a variety of mechanisms: exercise activates anti-inflammatory signaling pathways, such as inducing the release of anti-inflammatory cytokines (e.g., IL-10), and by inhibiting TNF-α and stimulating IL- 1ra exerting a direct anti-inflammatory effect (28); exercise helps to restore the homeostasis of the immune system and reduces the occurrence of excessive inflammatory responses (29, 30); moreover, by improving the function of the vascular endothelium, exercise can reduce the damage of inflammatory factors to the vascular wall, thus reducing the risk of atherosclerosis (31, 32).

Oxidative stress is a common pathophysiological phenomenon in AMI patients after surgery, which may lead to cardiomyocyte damage and dysfunction (33, 34). In this study, we found that exercise rehabilitation can effectively alleviate oxidative stress in AMI patients after PCI. Through exercise, the body can produce adaptive changes and enhance the antioxidant defense system, thereby reducing the generation of free radicals and the oxidative damage they trigger. This helps to restore the stability of the intra- and extracellular environment, reduce the damage of oxidative stress to cardiomyocytes, and thus protect cardiomyocytes and vascular endothelial cells and maintain the normal function of the cardiovascular system (35).

The enhancement of quality of life by exercise rehabilitation training is a direct reflection of its combined effect. By improving cardiac function and exercise endurance, and reducing inflammation and oxidative stress, exercise rehabilitation training positively affects patients at both the physiological and psychological levels. From the physiological point of view, the enhancement of cardiac function and exercise endurance significantly improves the mobility of patients, and at the same time effectively relieves somatic symptoms, enabling patients to cope with various needs in daily life more easily (15). From a psychological perspective, exercise prompts the body to secrete neurotransmitters such as endorphins, which improves the patients' emotional state, enhances psychological resilience, and enhances self-perception and social adaptability (36). This all-encompassing psychological adjustment not only improved patients' social functioning, but also significantly improved mental health.

The role of exercise rehabilitation training in reducing the incidence of adverse cardiovascular events should also not be overlooked. Exercise improves cardiac function, regulates the levels of inflammatory factors and oxidative stress, stabilizes coronary atherosclerotic plaques, and reduces the risk of plaque rupture and thrombosis (37, 38); exercise also lowers blood pressure and lipids, controls body weight, and improves glycemic metabolism, minimizing the risk factors for cardiovascular disease and reducing the risk of adverse cardiovascular events (39, 40).

Despite the remarkable results of this study, some limitations remain. Participants and staff were not blinded, which may introduce performance bias; however, objective outcomes and blinded assessors minimized this risk. Such partial blinding is standard in exercise-based rehabilitation trials. The sample was only from one hospital, which may have geographical and population limitations and affect the generalizability of the results; the study period of 3 months is relatively short, and the long-term effects need to be further observed. Although the baseline data of the two groups of patients were comparable, confounding factors such as dietary structure, medication adherence, and lifestyle after discharge were not strictly controlled or tracked; future studies may consider expanding the sample size, strictly controlling for confounding factors and extending the follow-up time to further validate the long-term efficacy of exercise rehabilitation. In addition, the specific regulatory mechanisms of exercise rehabilitation on cardiac function, inflammatory factors and oxidative stress can also be explored in depth by combining with molecular biology techniques.

In conclusion, exercise rehabilitation can significantly improve several physiological function indexes, including cardiac function, inflammatory factor level and oxidative stress status, in AMI patients after PCI, and at the same time significantly improve the quality of life of patients. This suggests that this method has potential value in early postoperative rehabilitation.

## Data Availability

The original contributions presented in the study are included in the article/Supplementary Material, further inquiries can be directed to the corresponding author.
